# The Effect of Flexible Lightwand and Ultrasonography Combination on Complications of the Percutaneous Dilatational Tracheostomy Procedure

**DOI:** 10.7759/cureus.5232

**Published:** 2019-07-25

**Authors:** Omer Faruk Boran, Bora Bilal, Deniz Çakır, Hafize Oksuz, Fatih Mehmet Yazar, Maruf Boran, Yavuz Orak

**Affiliations:** 1 Anesthesiology and Reanimation, Kahramanmaraş Sütçü İmam University, Kahramanmaras, TUR; 2 General Surgery, Kahramanmaraş Sütçü İmam University, Kahramanmaras, TUR; 3 Internal Medicine Intensive Care Unit, Amasya Şerefeddin Sabuncuoğlu Hospital, Amasya, TUR

**Keywords:** percutaneous dilatational tracheostomy, flexible lightwand, ultrasound guided

## Abstract

The aim of this study was to evaluate the effect of the flexible lightwand and ultrasonography (USG) combination on reducing the complications in percutaneous dilatational tracheostomy (PDT) opened with the forceps dilatation method. A retrospective examination was made of 138 patients between January 2014 and December 2018. Before starting to process, the anatomic structures of the patients were visualized with USG and the tracheostomy area was marked. Sedation and local anesthesia were applied to patients before the procedure, then the percutaneous tracheostomy was performed using the Griggs technique after confirmation of the tracheostomy localization defined with USG using the transillumination method with a flexible lightwand within an endotracheal tube. Complications that developed associated with the procedure were recorded. The mean age of the patients was 59.1±22.0 years and the mean length of stay in the intensive care unit was 42.3±35.5 days (range, 11-207 days). Overall, complications developed in 22 (15.6%) patients, of which 10.7% were early complications (1.4% related to the tube, 5.8% minor and 3.5% major complications). Tube- related complications were seen to develop in two patients. In the evaluation of the early minor complications, the most frequently seen complication was minor bleeding in 5.8% of the patients. No major vessel bleeding was determined in any patient in the early or late period. Of the late complications, the infection was seen to develop in four (2.8%) patients and stenosis in three (2.1%). The combination of flexible lightwand and USG in the PDT procedure minimized tube-related complications and contributed to the prevention of bleeding complications.

## Introduction

Patients applied with endotracheal intubation in Intensive Care Units (ICU) may remain attached to a mechanical ventilator for a long time. Prolonged endotracheal intubation can result in complications such as laryngeal damage, vocal cord paralysis, glottic and subglottic stenosis, infectious complications, and tracheal damage (tracheomalacia, tracheal dilatation, and tracheal stenosis) [1]. To reduce these complications associated with prolonged endotracheal intubation and to maintain the airway in ICU and prevent complications which may be associated with intubation, tracheostomy has started to be widely used in recent years to reduce dead space volume, reduce airway resistance, and provide patient comfort in the period of weaning off from the mechanical ventilator. Tracheostomies were first used in 3600 BC and became standardized in 1909 [2].

In 1955, Sheldon et al. defined the percutaneous dilatation tracheostomy (PDT) method, but this technique was abandoned after a short time because of serious complications such as carotid artery and esophagus injuries [3]. The technique was modified with the Seldinger method in 1969 and was modified again with the use of dilators by Ciaglia in 1985 [4]. Although studies have reported the applicability of this technique and that it is safer and more advantageous in respect of perioperative and postoperative complications, the PDT procedure has various early and late-stage complications.

Primary of these complications are minor or major bleeds, and life-threatening events such as aspiration, pneumothorax, infection, and esophagus injury [5]. To overcome these complications, in recent years the procedure has been combined with ultrasonography (USG), bronchoscopy, and transillumination methods using various illuminating devices [6-7]. USG is preferred because of the advantages of ease of application, it can be repeated when necessary and can be applied at the bedside, and by allowing the objective visualization of anatomic structures, it reduces the development of complications [6-8].

The transillumination PDT method was first defined by Addas et al., and in addition to the differentiation of vascular structures in particular, because of advantages such as determining the localization of the intubation tube, it has the advantage of preventing complications which may not be able to be determined with USG, such as bursting of the tube cuff, and the needle passing from the tube or the guidewire passing from the “Murphy eye” [9]. Bronchoscopy is another method used for this purpose [7-9]. This method was first described by Marelli, and although it provides advantages, especially in tracheal posterior wall injuries [10], the widespread use of the method has been limited by disadvantages such as the lengthy set-up of the device, increased costs, and that it may cause airway obstruction [7].

The aim of this study was to evaluate the effect of the flexible lightwand+USG combination on reducing the complications in PDT opened with the forceps dilatation method.

## Materials and methods

Setting and patients

Approval for the study was granted by the Kahramanmaras Sutcu Imam University, Faculty of Medicine Clinical Researches Ethics Committee (approval no: 2019/04/11, dated:06.03.2019). A retrospective examination was made of 161 adult patients who underwent percutaneous tracheostomy with the forceps dilatation technique (Griggs technique) between January 2014 and December 2018 in a 20-bed capacity tertiary level ICU, which admits mean 1200 patients per year. All the patients had prolonged intubation, upper airway obstruction, active partial thromboplastin time and prothrombin time 1.5-fold less than the control time, and thrombocyte count more than 50,000/mm [3]. No patients had a goiter, a history of neck surgery, or infection in the neck.

Exclusion criteria were defined as a history of surgery in the tracheostomy region, the development of local infection during the procedure, short neck and large thyroid tissue, cervical vertebra fracture, restriction in neck extension, morbid obesity, age less than 18 years, or non-application of PDT. All the procedures were performed under elective conditions and before opening the tracheostomy, first-degree relatives of the patient were informed about the procedures and written informed consent was obtained. Patient data were obtained from archived patient files and computer records, patient discharge summaries, ICU daily follow-up forms, operating notes, and observation records. A total of 23 patients were excluded from the study; eight could not be contacted after discharge and 15 had incomplete data. Thus, a total of 138 patients were included in the study for evaluation. 

Procedures

Percutaneous tracheostomies opened in our clinic are performed by a team comprising a specialist physician with approximately 20 years of experience, an assistant physician, and a nurse. The PDT procedure was performed as described below, using a Portex PDT set (Portex, Hythe, Kent, UK). Enteral nutrition of the patients was cut 6 hours before the procedure. Electrocardiography and pulse oximetry were applied throughout the procedure and noninvasive artery pressure was monitored with respiratory end carbon dioxide pressure.

First, visualization of the trachea was obtained with USG in the transverse plane from the mandible to the upper edge of the sternum, then with scanning in the sagittal plane, the thyroid, cricoid cartilage, vascular structures, vocal cords, and cartilage rings of the trachea were visualized. The 2nd and 3rd cartilage rings where the tracheostomy was to be applied were identified, and the site of the incision and needle entry between them was marked. The entry depth was determined by measuring the distance between the skin and the internal anterior wall of the trachea.

Then the distance advanced by the flexible lightwand over an endotracheal tube (ETT) (Beybi; Well Lead Medical Co, Guangzhou, China), as described by Zhao et al. was measured and marked for each patient [8]. The flexible lightwand used is accepted as a source of red LED cold light that can provide more than 400 lm brightness, which does not damage the tracheal mucosa. With the external diameter of approximately 3.4 mm of the light source used and the inner diameter of a minimum 8 mm ETTs for males and 7.0 mm ETTs for females is used, it was considered that the effect of the flexible lightwand on the ventilation resistance of the patient was minimal. Before the procedure, sedation and muscle relaxation were provided with the intravenous administration of fentanyl 1-2 μg kg^-1^, midazolam 0.2 mg kg^-1^ and rocuronium 0.5-1 mg kg^-1^, then positive pressure mechanical ventilation was applied with 100% oxygen.

The patient was positioned supine with support below the shoulders and the head brought into extension. The neck region was wiped with an antiseptic solution and sterile drapes, leaving the procedure area exposed. The endotracheal tube balloon was deflated and after pulling the tube to below the vocal cords, the tube balloon was re-inflated. By palpating the 2nd and 3rd spaces of the tracheal cartilage, local anesthesia of 2% lidocaine (2-3 mL) containing 1/100,000 adrenalin was applied to the area where the procedure was to be performed, then after confirming the tracheostomy localization determined with USG, a 14G needle was entered into the tracheal lumen using the translumination method with the flexible lightwand placed within the ETT as far as the marked section. 

After placement of the guidewire in the tracheal lumen, the needle was withdrawn, and making a horizontal incision from the edges of the wire with a scalpel, the skin, subcutaneous tissue, and trachea were expanded under the guidance of an 8F dilatator with the Seldinger method, then an appropriately sized tracheostomy tube was placed in the trachea. The cuff of the tracheostomy cannula was inflated and after listening to the respiratory sounds and cleaning around the tracheostomy tube, it was wrapped in the sterile sponge and the endotracheal tube was removed. A pulmonary radiograph was taken and the patient was closely monitored for 24 hours in respect of early complications.

The procedure time and the clinician performing the procedure together with data related to early complications were recorded on observation forms by the ICU nurse. Complications which may develop in the early period include bursting of the cuff, the needle passing from the tube (tube-origin), bleeding, aspiration, subcutaneous emphysema, pneumothorax, and air embolism, tracheal posterior wall damage, oesophageal injury, stoma infection complications (early local complications), stenosis, and late-stage infection. The decision for weaning and decannulation of the patients was made by evaluating parameters such as sufficient vital capacity, ability to protect the airway, absence of or reduced tracheobronchial secretions, and spontaneous respiration attempts.

For each patient, a record was made of age, gender, primary diagnoses, and comorbidities requiring mechanical ventilation, GCS values, length of stay in ICU, total duration of mechanical ventilation, discharge status (discharged healthy or with silver cannula or home-type mechanical ventilator, or exitus), early local complications of tube origin that developed after the intervention, late-stage complications (bleeding, subcutaneous emphysema, pneumothorax incorrect passage, hypotension, hypoxia, mortality), systolic and diastolic blood pressure values, heart rate, peripheral oxygen saturation values, and the duration of the intervention.

Statistical analysis

Data obtained in the study were analyzed statistically using SPSS for Windows in 15.0 software (Statistical Package for the Social Sciences, Armonk, NY, USA). Results were stated as number (n) and percentage (%) or mean ± standard deviation values.

## Results

A total of 138 patients admitted to ICU for various reasons were applied with a percutaneous dilatational tracheostomy with the flexible lightwand + USG combination. The reasons for admittance to ICU are given in Table 1. The patients comprised 71.7% males and 28.3% females with a mean age of 59.1±22.0 years. Mortality developed in 58.0% of all the patients included in the study and the highest mortality rate was seen in the diagnostic group of ischemic or hemorrhagic cerebral damage (Table 1).

**Table 1:** Demographic features and medical condition of the study population

Variables	Mean	SD
Age (years)	77.79	7.35
GCS	5.81	2.37
Days ventilated prior to tracheostomy (days)	12.98	8.81
ICU hospitalization day	42.3	35.5
Variables	n	%
Gender (male)	99	71.7
Mortality in ICU	80	58
Indication for tracheostomy	138	100
Infections	5	3.6
Respiratory failure	18	13
Cardiovascular	17	12.3
Surgical	19	13.7
Neurological	63	45.8
Miscellaneous	16	11.6
GCS: Glasgow Coma Score; ICU: intensive care unit; SD: standart deviation		

The mean length of stay in ICU of all the patients applied with a tracheostomy was 42.3±35.5 days (range, 11-207 days). The mean GCS, duration of intubation, and the total duration of mechanical ventilation are presented in Table 1. The PDT intervention was made at the earliest on the first day of admission to ICU and at the latest on the 37th day. The mean time to tracheostomy was determined as 12.98±8.81 days from the time of admission to ICU. Overall, complications developed in 22 (15.4%) patients, of which 10.5% were early complications (1.4% related to the tube, 5.6% minor and 3.5% major complications).

Tube-related complications were seen to develop in two patients, one of which was the guidewire passing into the “Murphy eye” and the other was tube cuff rupture. In the evaluation of the early minor complications, the most frequently seen complication was minor bleeding in 5.8% of the patients. All the cases of minor bleeding were stopped with pressure applied then a gauze dressing soaked with adrenalin or tranexamic acid. No major vessel bleeding was determined in any patient in the early or late period.

Early stage major complications were observed to be aspiration in 2 (1.4%) patients, emphysema in 1 (0.7%), emphysema+pneumothorax in 1 (0.7%), and mortality in 1 (0.7%). The complications that developed as a result of the PDT procedure are presented in Table 2. Of the late complications, the infection was seen to develop in 4 (2.8%) patients and stenosis in 3 (2.1%).

**Table 2:** Incidence of tracheostomy complications

Type of complication	n (%)
Early	15 (10.5)
Tube related	2 (1.4)
Guidewire passing into the “Murphy eye	1 (0.7)
Cuff rupture	1 (0.7)
Minor complications (minor bleeding controlled by local measures, not requiring reexploration or transfusion)	8 (5.6)
Major complications	5 (3.5)
Subcutaneous emphysema	1 (0.7)
Aspiration	2 (1.4)
Subcutaneous emphysema+pneumothorax	1 (0.7)
Mortality	1 (0.7)
Late	7 (4.9)
Wound infection	4 (2.8)
Tracheal stenosis	3 (2.1)

On discharge of the patients, 8 (5.8%) had spontaneous respiration with the closure of the tracheostomy, 38 (27.5%) were discharged with spontaneous respiration with a silver cannula, and 12 (8.7%) were discharged with home-type mechanical ventilation support.

## Discussion

In cases requiring long-term mechanical ventilation support in ICU, tracheostomy is performed to reduce endotracheal intubation complications to a minimum such as vocal cord paralysis, glottic and subglottic stenosis, to reduce dead space volume and airway resistance, and to provide patient comfort [2]. In the last 30 years, PDT has started to be applied instead of surgical tracheostomy as it can be opened by a doctor without surgical training, reduces costs and transport risks as it can be performed at the bedside, and there is less tissue damage, less stoma infection, and has better cosmetic results [4,7].

Although PDT has lower complication rates than surgical tracheostomy, in the classic method the ETT balloon is deflated and the tube is pulled up below the vocal cords to avoid tube-related complications [7-8]. However, although this prevents tube-related complications, there is the risk of involuntary extubation [8,11]. Therefore, to be able to overcome tube-related complications, the procedure has been modified with some methods such as FOB [6], flexible lightwand [8], and inflation of the ETT cuff with saline [12]. Each of these methods has different limitations. For example, while the use of FOB prevents tube-related complications, it also reduces tracheal posterior wall damage to a minimum, which is one of the most frequently seen complications of the procedure. However, a worsening of ventilation leading to hypercarbia and/or hypoxia seen in approximately 16.7% of patients is the most significant disadvantage [13]. Shankar et al. filled the ETT cuff with saline following the determination of the anatomic structures with USG and emphasized that damage of the tube cuff could be determined [12]. Nevertheless, the risk of aspiration that can emerge as a result of cuff rupture is the most significant disadvantage of this technique [12].

In some studies, USG has been used alone. Despite the clear identification of anatomic and vascular structures with USG, no effect on ETT complications has been reported [12,14]. Finally, a recent study has recommended the use of lightwand, which is a simple, cost-effective method used in difficult airway management, to prevent tube-related complications in particular. However, this technique is ineffective in the prevention of complications related to vascular structures [8]. To the best of our knowledge, the current study is the first to have combined USG and lightwand to prevent PDT-related complications.

PDT complications can be classified under two headings as early and late complications. Within the early-stage complications, tube-related complications can be evaluated as minor and major. Tube-related complications that make the procedure technically more difficult and can affect patient safety can be evaluated as significant complications. To prevent this in the classic method, the cuff is deflated and the tube is pulled up to 17 cm in males and 15 cm in females, then the cuff is re-inflated. However, although cuff rupture can be prevented with this method, there is a risk of extubation [8].

Ambesh et al. reported rates of cuff puncture of 6.6% and the incidence of unwanted extubation as 3.3% [15]. Karımpour et al. reported cuff puncture as 1.1% and airway loss as 1.1% [16]. In the current study, accidental extubation did not develop in any patient, and tube-related complications were observed in 2 (1.4%) patients. One (0.7%) of these tube-related complications was the guidewire having entered the Murphy eye, and the other (0.7%) was cuff rupture. In comparison with reports in the literature, the rate of tube-related complications in the current study was extremely low. This was attributed to relatively better prediction of the localization of the distal end of the tube with the flexible lightwand method.

In literature, the most common complication related to PDT has been reported to be bleeding (0%-36%) but the relevant data in studies are extremely heterogeneous. In some studies, bleeding is classified as minor and major bleeds (requiring blood or blood product replacement and/or medical or surgical hemostasis), while in other studies, only major bleeds are accepted as bleeding complications [16]. In a study by Diaz-Reganon et al. in which 800 PDT procedures were evaluated, bleeding was reported to have developed in 13 (1.6%) patients [17]. However, Staffer reported that bleeding developed in 36% of patients [18]. In the current study, bleeding complications were separated as minor and major bleeds. No major bleed developed in any patient and minor bleeds were seen to develop in 5.8%. All of these bleeds were stopped with local pressure and dressings applied of gauze soaked in adrenalin or tranexamic acid. In literature, major bleeds have been reported to occur in 0%-4.2% of patients applied with PDT [7]. The most important reason that no major bleed developed in any of the current study patients is thought to be due to the evaluation of the vascular structures with USG and visualization of the vascular structures with transillumination.

Emphysema developed in 1 (0.7%) patient and emphysema+pneumothorax in 1 (0.7%) patient in the current study. Both of these complications developed during the first 50 procedures applied, and while the patient with emphysema+pneumothorax was treated with tube thoracostomy, the patient who developed emphysema only was observed to have spontaneous resorption at the end of follow-up. In literature it has been emphasized that emphysema and pneumothorax complications are one of the most common complications occurring in inexperienced hands, caused by a highly inclined entry of the needle, resulting in catching the trachea. Karimpour et al. applied PDT with the Griggs technique and reported that emphysema developed in 2 (1.1%) patients and pneumothorax in 1 (0.5%) [16]. Goldenberg et al. also reported that emphysema developed in 1 patient and pneumothorax in 1 [19]. When the current study results are evaluated with respect to emphysema and pneumothorax complications, they can be seen to be consistent with previous findings in the literature. When the practitioner fears pneumothorax and emphysema complications and advances the guide needle very vertically, posterior wall damage may occur [16]. No complications related to the posterior wall were observed in the current study.

Late-stage complications of tracheostomy include tracheal stenosis, tracheal-oesophageal fistula, and tracheocutaneous fistula. In some studies, late complication rates have been reported to vary between 2% and 60% [20-21]. In the current study, the late complication rate was 4.9% and the rate of tracheal stricture was 2.1%. In some studies, tracheal stricture has been reported as 1.6%-9.1%, with the narrowing of the tracheal lumen caused by fibrosis or granulation tissue, although it has been emphasized that this is of clinical importance in only 3%-12% of cases [16,21-23]. In a study by Karimpour et al. PDT was applied using the Griggs technique to 183 patients and tracheal stricture was reported to have developed in 3 (1.8%) patients [16]. In another study by Wagner et al. tracheal stricture developed in 8 (9.1%) patients of a series of 88 applied with percutaneous tracheostomy [23]. In the current study, the results related to tracheal stricture were seen to be consistent with the literature. However, 8 patients were excluded from the study as they could not be contacted after discharge, and therefore no information could be obtained about late-stage complications which potentially could have developed in those patients.

The most important limitation of this study was that because of the retrospective design, it was dependent on the quality of documentation kept in the ICU. However, for the last 10 years, the patient records in our clinic have been kept in the computerized system and in great detail on the nurse observation forms. Therefore, data loss was considered to be at a minimum level. Nevertheless, problems were experienced in obtaining the data of some discharged patients, so these patients were excluded from the study and no information could be obtained related to late anatomic complications which could develop in these patients. The absence of a control group was another limitation, but it was attempted to use reports in the literature as a reference in this respect.

## Conclusions

The effect of the flexible lightwand and USG combination used during the PDT procedure was evaluated with respect to reducing complications of tracheostomy. The results demonstrated that tube-related complications were minimized due to the use of flexible lightwand and the transillumination combination contributed to minimizing bleeding complications.
